# The Effects of Soil Cover Thickness on Leaf Functional Traits of Vine Plants in Mining Areas Depend on Soil Enzyme Activities and Nutrient Cycling

**DOI:** 10.3390/plants14142225

**Published:** 2025-07-18

**Authors:** Ren Liu, Yun Sun, Zongming Cai, Ping He, Yunxia Song, Longhua Yu, Huacong Zhang, Yueqiao Li

**Affiliations:** 1 Experimental Center of Subtropical Forestry, Chinese Academy of Forestry, Xinyu 336600, China; liuren951106@caf.ac.cn (R.L.); m15707933437@163.com (Y.S.); cafczm1998@163.com (Z.C.); peacejx@163.com (P.H.); yunxsoong@163.com (Y.S.); yulonghuapuesue@126.com (L.Y.); zhang718736170@163.com (H.Z.); 2 Jiangxi Provincial Key Laboratory of Biodiversity Conservation and Resource Utilization, Xinyu 336600, China

**Keywords:** leaf functional traits, nutrient cycling, soil cover thickness, soil enzyme activity, vine plants

## Abstract

Understanding the interplay between plant leaf functional traits and plant and soil factors under different soil thicknesses is significant for quantifying the interaction between plant growth and the environment. However, in the context of ecological restoration of vegetation in mining areas, there has been a lot of research on trees, shrubs, and grasses, but the characteristics and correlations of leaf functional traits of vines have not been fully studied to a large extent. Here, we report the differences in leaf functional traits of six vine plants (*Parthenocissus quinquefolia*, *Pueraria lobata*, *Hedera nepalensis*, *Campsis grandiflora*, *Mucuna sempervirens*, and *Parthenocissus tricuspidata*) with distinct growth forms in different soil cover thicknesses (20 cm, 40 cm, and 60 cm). In addition, soil factor indicators under different soil cover thicknesses were measured to elucidate the linkages between leaf functional traits of vine plants and soil factors. We found that *P. lobata* showed a resource acquisition strategy, while *H. nepalensis* demonstrated a resource conservation strategy. *C. grandiflora* and *P. tricuspidata* shifted toward more conservative resource allocation strategies as the soil cover thickness increased, whereas *M. sempervirens* showed the opposite trend. In the plant trait–trait relationships, there were synergistic associations between specific leaf area (SLA) and leaf nitrogen content (LNC); leaf moisture content (LMC) and leaf nitrogen-to-phosphorus ratio (LN/P); and leaf specific dry weight (LSW), leaf succulence degree (LSD), and leaf dry matter content (LDMC). Trade-offs were observed between SLA and LSW, LSD, and LDMC; between leaf phosphorus content (LPC) and LN/P; and between LMC, LSW, and LDMC. In the plant trait–environment relationships, soil nutrients (pH, soil total phosphorus content (STP), and soil ammonium nitrogen content (SAN)) and soil enzyme activities (cellulase (CB), leucine aminopeptidase (LAP), enzyme C/N activity ratio, and enzyme N/P activity ratio) were identified as the primary drivers of variation in leaf functional traits. Interestingly, nitrogen deficiency constrained the growth of vine plants in the mining area. Our study revealed that the responses of leaf functional traits of different vines under different soil thicknesses have significant species specificity, and each vine shows different resource acquisition and conservation strategies. Furthermore, soil cover thickness primarily influences plant functional traits by directly affecting soil enzyme activities and nutrients. However, the pathways through which soil thickness impacts these traits differ among various functional traits. Our findings provide a theoretical basis and practical reference for selecting vine plants and optimizing soil cover techniques for ecological restoration in mining areas.

## 1. Introduction

Ecological restoration in mining areas has long been a focal point of research in the fields of environmental science and mining engineering [1]. During mineral resource extraction, large-scale soil stripping often occurs, leading to the destruction of soil structure, nutrient loss, and the degradation of ecological functions [2,3]. To restore ecological functions in mining areas, scientifically sound land reclamation and vegetation restoration have become key strategies for environmental management in these regions [4]. Soil cover techniques for land reclamation in mining areas are crucial for restoring the ecological environment and improving land quality. Studies have shown that soil cover thickness significantly influences nutrient cycling, greenhouse gas emissions, and the composition and diversity of soil microorganisms [5,6]. The thickness of the cover layer not only directly affects the physical properties of the soil, such as structure, porosity, and water retention capacity [7], but also indirectly impacts its chemical properties and biological activity, including enzyme activities and nutrient cycling [8]. Proper soil cover measures can prevent soil erosion, improve soil quality, promote plant growth, and provide long-term sustainable value for land use in mining areas [9,10]. Therefore, the rational design and application of soil cover is an indispensable component of mining area environmental management. Through scientifically designed soil covering techniques, the ecological environment of mining areas can be effectively restored, providing a stable foundation for subsequent agricultural, forestry, or other uses.

The functional traits of plants are physiological or morphological characteristics that evolve through natural selection, allowing plants to adapt to their environment and enhance their survival capabilities. These traits reflect the intricate interplay between plant species and their habitat, as well as interactions with competitors and other organisms [11]. Leaves, as the primary organs for photosynthesis and transpiration, are the most sensitive to environmental changes. Through long-term adaptation to the environment, plants have developed an optimal combination of traits for environmental suitability [12]. Studies have shown that leaf morphological traits, such as specific leaf area (SLA), leaf shape factor (LSF), and leaf dry matter content (LDMC), as well as chemical traits, including leaf nitrogen content (LNC), leaf phosphorus content (LPC), and their quantitative characteristics, play crucial roles in determining plant physiological functions and biogeochemical cycles [13,14]. Throughout their growth and development, plants do not respond to environmental factors through a single trait alone; rather, their responses are the integrated outcome of the coordinated actions of multiple traits. Leaf traits in plants are often interrelated and mutually influential. For instance, there is a close, significantly negative correlation between LNC and LDMC, and a significantly positive correlation between LNC and LPC. Additionally, leaf specific weight (LSW) is positively correlated with LDMC [15]. Moreover, soil is a pivotal determinant influencing plant leaf functional traits. Li S et al. [12] observed that soil organic carbon, pH, and soil moisture content were critical factors influencing shrub leaf trait variations, while available phosphorus and soil organic carbon were the primary factors influencing herbaceous plant leaf traits. Song Y et al. [16] found that the soil carbon-to-nitrogen ratio, total soil calcium, soil moisture content, available phosphorus, and the carbon-to-calcium ratio in soil showed strong correlations with leaf functional traits in *Zanthoxylum planispinum* ‘dintanensis’, indicating a significant impact of soil chemical stoichiometry on leaf functional traits. Furthermore, soil enzyme activities are intrinsically connected to soil fertility and plant growth status [17,18]. Despite numerous studies revealing the broad impact of soil properties on plant functional traits, investigations into the interplay between leaf functional traits and soil enzyme activities in mining areas remains insufficient. Therefore, in-depth research on the relationship between plant leaf functional traits and soil enzyme activities in mining areas can not only reveal the coupling between resource allocation strategies and underground nutrient supply during vegetation restoration but also provide a theoretical foundation for selecting plant species suited to mining areas, with significant ecological value and practical implications.

Soil degradation and nutrient loss in mining areas interfere with nutrient cycling processes, subsequently affecting plant growth and ecosystem restoration. Vine plants, due to their strong adaptability and favorable growth characteristics, can maintain species diversity and increase vegetation cover [19], making them important for ecological restoration in mining areas. Research on the functional traits of vine plant leaves can, to some extent, quantify the interaction between plant growth and the environment, further exploring the growth strategies of these plants. However, existing studies mainly focused on the trait–trait relationships and trait–environment relationships of trees, shrubs, and grasses [20,21,22,23], with limited research on the ecological strategies and trait trade-offs of vine plants in artificial soil-covered habitats in mining areas with high and steep rock walls.

Therefore, this study selects six vine plant species (*Parthenocissus quinquefolia*, *Pueraria lobata*, *Hedera nepalensis*, *Campsis grandiflora*, *Mucuna sempervirens*, and *Parthenocissus tricuspidata*) as the research subjects. An in situ experiment with different soil cover thicknesses is conducted at the bottom of steep rock walls in a mining area. By measuring the vine plant leaf functional traits and key soil factor indicators, intending to explore the differences in leaf functional traits and soil factors across varying soil cover thicknesses, we investigate the adaptation of vine plants to various soil cover thicknesses (20 cm, 40 cm, and 60 cm), and analyze the trait–trait and trait–environment relationships. Additionally, the study analyzes how leaf functional trait changes in vine plants respond to soil cover thickness. This research is structured around the following central inquiries: (i) How does soil cover thickness interact with leaf functional traits and soil factors? (ii) Are there trade-offs or synergies among leaf functional traits, and how do traits relate to the environment? (iii) How does soil cover thickness directly or indirectly affect leaf functional traits? We hypothesize that: (i) the effect of soil cover thickness on leaf functional traits and soil factors differs across vine plant species; (ii) the trait–trait and trait–environment relationships of vine plants vary; and (iii) soil cover thickness may indirectly influence leaf functional traits via soil enzyme activities and nutrient availability. The results of this study will help to clarify the differentiation patterns of leaf traits and soil properties across different soil cover thicknesses and reveal the regulatory mechanisms of soil enzyme activities and nutrient dynamics in the control of leaf traits by soil cover thickness. Ultimately, this will contribute to understanding the adaptive strategies of vine plants to environmental changes, providing a theoretical basis and practical reference for the selection of vine plants and the optimization of soil cover techniques for ecological restoration projects in mining areas with high and steep rock walls.

## 2. Results

### 2.1. Differences in Leaf Functional Traits of Vines Under Different Soil Cover Thickness Treatments

**Figure 1:**
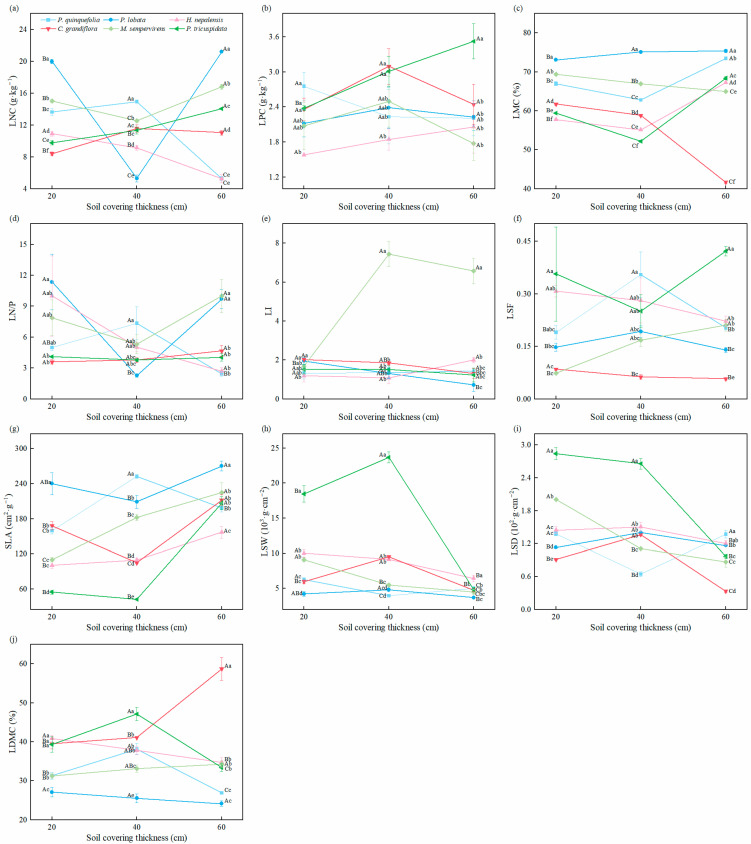
Leaf functional traits of vines under different soil cover thickness treatments. (**a**) LNC, Leaf nitrogen content; (**b**) LPC, Leaf phosphorus content; (**c**) LMC, Leaf moisture content; (**d**) LN/P, Leaf nitrogen/phosphorus; (**e**) LI, Leaf shape index; (**f**) LSF, Leaf shape factor; (**g**) SLA, Specific leaf area; (**h**) LSW, Leaf specific weight; (**i**) LSD, Leaf succulence degree; (**j**) LDMC, Leaf dry matter content.The uppercase letters indicate significant differences for the same vine type between different soil cover thicknesses (*p* < 0.05). The lowercase letters indicate that there were significant differences among different types of vines in the same soil cover thickness (*p* < 0.05).

Based on Figure 1, it was observed that soil cover thickness and its interaction with vegetation types had significant effects on various leaf functional traits, with the exception of LPC and LSF. Soil cover thickness significantly influenced most leaf functional traits, with the exception of LPC and LSF. At a 20 cm soil cover thickness, significant differences in LMC were found among different vine plants (*p* < 0.05), with the order of LMC being: *P. lobata* > *M. sempervirens* > *P. quinquefolia* > *C. grandiflora* > *H. nepalensis* > *P. tricuspidata*. The LPC of *P. quinquefolia* (2.75 g·kg^−1^) was significantly higher than that of the other vine plants (*p* < 0.05), while the LPC of *H. nepalensis* (1.58 g·kg^−1^) was the lowest. *C. grandiflora* exhibited low values of LNC (8.40 g·kg^−1^) and LN/P (3.60). The SLA of *P. lobata* (240.09 cm^2^·g^−1^) was significantly higher than that of the other five vine plants, although its LSW and LDMC (27.10%) were significantly lower. By contrast, *P. tricuspidata* showed a significantly lower SLA (54.63 cm^2^·g^−1^) than the other vine plants (*p* < 0.05), but its LSW, LSD, and LDMC were higher than those of the other vine plants at both 20 cm and 40 cm soil cover thicknesses (*p* < 0.05).

At a 40 cm soil cover thickness, significant differences in LMC were observed among the different vine plants (*p* < 0.05), with the order of LMC being: *P. lobata* > *M. sempervirens* > *P. quinquefolia* > *C. grandiflora* > *H. nepalensis* > *P. tricuspidata*. The LNC (14.95 g·kg^−1^), LN/P (7.35), and SLA (252.41 cm^2^·g^−1^) of *P. quinquefolia* were significantly higher than those of the other vine plants (*p* < 0.05). *H. nepalensis* and *C. grandiflora* exhibited lower values of certain leaf traits compared to the other vine plants at all three soil cover thicknesses, although the LPC of *C. grandiflora* (3.10 g·kg^−1^) was the highest. *M. sempervirens* had a significantly higher LI (7.44) than the other five vine plants (*p* < 0.05).

At a 60 cm soil cover thickness, significant differences in LMC were again observed among the vine plants (*p* < 0.05), with the order of LMC being: *P. lobata* > *P. quinquefolia* > *P. tricuspidata* > *H. nepalensis* > *M. sempervirens* > *C. grandiflora*. *P. lobata* exhibited a significantly higher LNC (21.19 g·kg^−1^) and SLA (270.10 cm^2^·g^−1^) compared to the other vine plants, while its LI and LSW were significantly lower (*p* < 0.05). *C. grandiflora* had the highest LDMC (58.77%) among all of the vine plants, while *M. sempervirens* had the highest LN/P (10.00) and LI (6.58). The LPC of *P. tricuspidata* (3.52 g·kg^−1^) and the LSF of *P. tricuspidata* were significantly higher than those of the other vine plants (*p* < 0.05).

Furthermore, it was found that the LN/P values for all vine plants across different soil cover thicknesses were consistently below 14. *P. lobata* had the highest LMC, LNC, and LN/P across all three soil cover thicknesses compared to the other vine plants. The LI of *P. lobata* decreased with increasing soil cover thickness. Both the LNC and LN/P of *H. nepalensis* decreased as soil cover thickness increased. For *C. grandiflora*, the LMC, LI, and LSF decreased with increased soil cover thickness, whereas the LNC and LDMC showed the opposite trend. For *M. sempervirens*, the LI, LSF, SLA, and LDMC increased with soil cover thickness, while the LMC, LSW, and LSD decreased. In *P. tricuspidata*, the LNC, LPC, and SLA increased significantly with increasing soil cover thickness, while the LSD decreased with increasing soil cover thickness.

### 2.2. The Effect of Vines on Soil Physical and Chemical Properties of Different Soil Cover Thicknesses in the Mining Area

**Figure 2:**
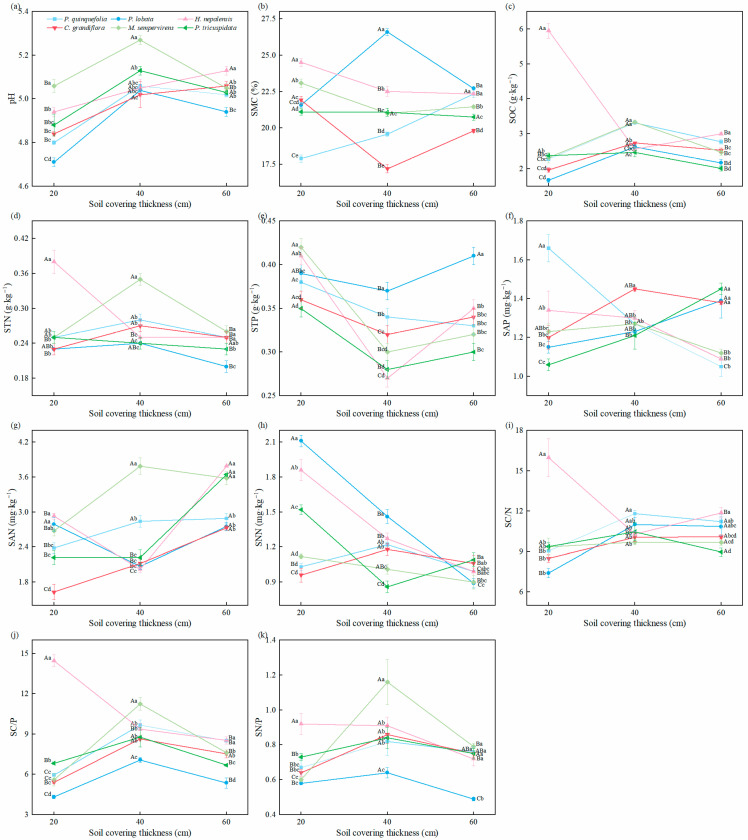
The effect of vines on soil physical and chemical properties of different soil cover thicknesses in the mining area. (**a**) pH; (**b**) SMC, Soil moisture content; (**c**) SOC, Soil organic carbon; (**d**) STN, Soil total nitrogen; (**e**) STP, Soil total phosphorus; (**f**) SAP, Soil available phosphorus; (**g**) SAN, Soil ammonium nitrogen; (**h**) SNN, Soil nitrate nitrogen; (**i**) SC/N, Soil organic carbon/total nitrogen; (**j**) SC/P, Soil organic carbon/total phosphorus; (**k**) SN/P, Soil total nitrogen/total phosphorus. Different uppercase letters in the same column indicate significant differences between different soil layers of the same plant (*p* < 0.05). Different lowercase letters in the same column indicate significant differences between six species in the same soil layer (*p* < 0.05).

Based on Figure 2, except for SAP and SC/N, soil physical and chemical properties were greatly influenced by the soil cover thickness, vine type, and their interaction. Soil pH values remained relatively stable for different vine species across varying soil cover thicknesses, ranging from 4.71 to 5.27, indicating slightly acidic conditions. At a 20 cm soil cover thickness, the SAP of *P. quinquefolia* (1.66 mg·kg^−1^) was greatly higher than that of the other vine plants (*p* < 0.05). *H. nepalensis* exhibited higher values of SMC, SOC, STN, SAN, SC/N, SC/P, and SN/P. The soil pH (5.06) and STP (0.42 g·kg^−1^) were higher when *M. sempervirens* was planted. By contrast, the STN was lower, but the SNN (2.11 mg·kg^−1^) was higher when *P. lobata* was planted.

At a 40 cm soil cover thickness, *P. quinquefolia* exhibited the highest SC/N. The SAP of *C. grandiflora* (1.45 mg·kg^−1^) and the soil pH, SOC (3.34 g·kg^−1^), STN, SAN, SC/P, and SN/P of *M. sempervirens* were the highest. For *P. lobata*, the SMC (26.59%), STP (0.37 g·kg^−1^), and SNN (1.46 mg·kg^−1^) were significantly higher than those of other vines (*p* < 0.05).

At a 60 cm soil cover thickness, *P. quinquefolia* exhibited a higher SMC, SC/P, and SN/P. When *P. lobata* was planted, the STP (0.41 g·kg^−1^) was significantly higher than in the other vine species (*p* < 0.05), while the SN/P was the lowest. *H. nepalensis* had the highest pH, SOC, SAN, SC/N, and SC/P, while *C. grandiflora* had the lowest SMC (19.81%). The SNN (1.09 mg·kg^−1^) and SAP (1.45 mg·kg^−1^) values were highest when it was planted.

For *P. quinquefolia*, the soil pH, SMC, SAN, and SC/N significantly increased with increasing soil cover thickness, while the STP and SAP showed decreasing trends. For *P. lobata*, the SAP and SC/N increased with soil cover thickness, while the SNN showed a decreasing trend. For *H. nepalensis*, the SMC, SAP, SNN, SC/N, SC/P, and SN/P decreased with increasing soil cover thickness, while the pH increased. For *C. grandiflora*, the pH, SAN, SC/N, and SC/P showed increasing trends. The SNN of *M. sempervirens* decreased with increasing soil cover thickness. For *P. tricuspidata*, the SAP and SAN increased with increasing soil cover thickness, while the SMC and STN decreased. The NAG and eN/P of *P. tricuspidata* showed decreasing trends.

### 2.3. The Effect of Vines on Soil Enzyme Activities and Stoichiometric Characteristics of Different Soil Cover Thicknesses in the Mining Area

**Figure 3:**
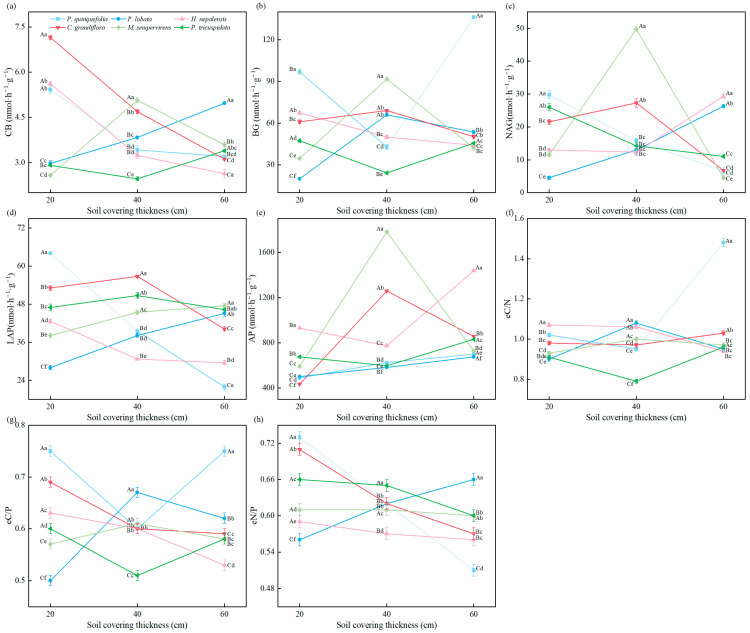
Soil enzyme activities and stoichiometric characteristics of vines under different soil cover thicknesses in the mining area. (**a**) CB, Cellulase; (**b**) BG, β-1,4-glucosidase; (**c**) NAG, N-acetyl-β-D-glucosaminidase; (**d**) LAP, Leucine aminopeptidase; (**e**) AP, Acid phosphatase; (**f**) eC/N, ln (BG + CB)/ln (NAG + LAP) enzyme C/N; (**g**) eC/P, ln (BG + CB)/ln (AP) enzyme C/P; (**h**) eN/P, ln (NAG + LAP)/ln (AP) enzyme N/P. Different uppercase letters in the same column indicate significant differences between different soil layers of the same plant (*p* < 0.05). Different lowercase letters in the same column indicate significant differences between six species in the same soil layer (*p* < 0.05).

Based on Figure 3, it was evident that soil enzyme activities and its stoichiometry were significantly influenced by soil cover thickness, the type of vine plants, and their interactions. At the 20 cm soil cover thickness, *P. quinquefolia* exhibited significantly higher BG (96.83 nmol·h^−1^·g^−1^), NAG (29.76 nmol·h^−1^·g^−1^), LAP (64.08 nmol·h^−1^·g^−1^), eC/P, and eN/P values compared to other vine plants (*p* < 0.05). *H. nepalensis* had the highest AP (930.49 nmol·h^−1^·g^−1^) and eC/N values, although its CB, BG, LAP, eC/N, eC/P, and eN/P values were relatively lower. By contrast, *P. lobata* exhibited lower BG, NAG, LAP, eC/N, eC/P, and eN/P values. *P. tricuspidata* had higher BG, NAG, eC/P, and eN/P values.

At the 40 cm soil cover thickness, *C. grandiflora* showed significantly higher LAP (56.80 nmol·h^−1^·g^−1^) and AP (1783.39 nmol·h^−1^·g^−1^) values, while *M. sempervirens* had the highest CB (5.07 nmol·h^−1^·g^−1^), BG (91.67 nmol·h^−1^·g^−1^), and NAG (49.74 nmol·h^−1^·g^−1^) values compared to other vine plants (*p* < 0.05). *P. lobata* exhibited the highest eC/N and eC/P values, while *P. tricuspidata* had the highest eN/P value.

At the 60 cm soil cover thickness, *P. quinquefolia* showed higher BG (136.09 nmol·h^−1^·g^−1^), eC/N, and eC/P values, although its LAP (21.92 nmol·h^−1^·g^−1^) and eN/P values were significantly lower than those of other vine plants (*p* < 0.05). *P. lobata* exhibited the highest CB and eN/P values, but the lowest AP (674.66 nmol·h^−1^·g^−1^) value. *H. nepalensis* showed the highest NAG (29.35 nmol·h^−1^·g^−1^) and AP (1438.64 nmol·h^−1^·g^−1^) values. *M. sempervirens* had the highest LAP (47.50 nmol·h^−1^·g^−1^) value but the lowest NAG (4.57 nmol·h^−1^·g^−1^) value.

When *P. quinquefolia* was planted, the CB, NAG, and LAP values showed decreasing trends, while the opposite trend was observed for *P. lobata*. For *C. grandiflora*, the CB and eC/P values decreased with increasing soil cover thickness, whereas the LAP and AP values of *M. sempervirens* increased significantly with thicker soil cover. *P. tricuspidata* showed a decreasing trend in NAG and eN/P values with increasing soil cover thickness.

### 2.4. The Relationship Between Leaf Functional Traits of Vines and Its Relationship with Soil Factors

**Figure 4:**
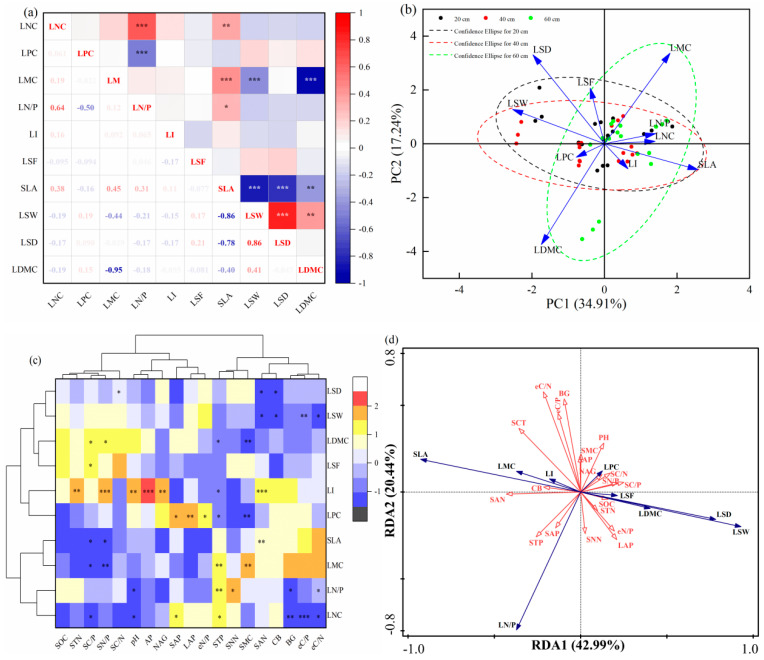
Relationships among leaf functional traits of vines. (**a**) Pearson’s correlation analysis of 10 leaf functional traits. (**b**) PCA of 10 leaf functional traits. The solid arrows indicate the direction and weighing of vectors that correspond to the 10 leaf functional traits. (**c**) Pearson’s correlation coefficients between soil factors and leaf functional traits. (**d**) Redundancy analysis between soil factors and leaf functional traits. Significance levels are categorized as follows: * *p* < 0.05; ** *p* < 0.01; *** *p* < 0.001. SCT, Soil covering thickness; LNC, Leaf nitrogen content; LPC, Leaf phosphorus content; LMC, Leaf moisture content; LN/P, Leaf nitrogen/phosphorus; LI, Leaf shape index; LSF, Leaf shape factor; SLA, Specific leaf area; LSW, Leaf specific weight; LSD, Leaf succulence degree; LDMC, Leaf dry matter content; SOC, Soil organic carbon; STN, Soil total nitrogen; STP, Soil total phosphorus; SMC, Soil moisture content; SAP, Soil available phosphorus; SAN, Soil ammonium nitrogen; SNN, Soil nitrate nitrogen; SC/N, Soil organic carbon/total nitrogen; SC/P, Soil organic carbon/total phosphorus; SN/P, Soil total nitrogen/total phosphorus; CB, Cellulase; BG, β-1,4-glucosidase; NAG, N-acetyl-β-D-glucosaminidase; LAP, Leucine aminopeptidase; AP, Acid phosphatase; eC/N, ln (BG + CB)/ln (NAG + LAP) enzyme C/N; eC/P, ln (BG + CB)/ln (AP) enzyme C/P; eN/P, ln (NAG + LAP)/ln (AP) enzyme N/P.

In Figure 4a, it could be observed that LNC showed a highly significant positive correlation with LN/P and SLA, while SLA had a highly significant negative correlation with LSW, LSD, and LDMC. By contrast, LPC was significantly negatively correlated with LN/P, while LN/P and LMC exhibited significantly positive correlations with SLA (Figure 4a). LMC exhibited a highly significant negative correlation with LSW and LDMC, while LSW was showed a highly significant positive correlation with LSD and LDMC. However, no significant correlations were detected between LI, LSF, and other functional traits (Figure 4a). Principal component axes 1 (PC1) and axes 2 (PC2) explained 52.15% of the total trait variation, capturing the majority of the functional trait variability (Figure 4b). The correlations among functional traits derived from principal component analysis were consistent with those from the Pearson’s correlation test.

Figure 4c showed that LNC was significantly positively correlated with SAP and STP and significantly negatively correlated with SC/P, pH, BG, eC/P, and eC/N. LN/P was significantly positively correlated with SNN and STP and significantly negatively correlated with pH, BG, and eC/N. LPC was significantly positively correlated with SAP, LAP, and eN/P and significantly negatively correlated with STP and SMC (Figure 4c). LSD was significantly negatively correlated with SC/N, SAN, and CB (Figure 4c), while LSW was significantly negatively correlated with SAN, CB, eC/P, and eC/N. LDMC was significantly positively correlated with SC/P and SN/P and significantly negatively correlated with STP and SMC; LMC showed the opposite pattern (Figure 4c). Additionally, LSF was positively correlated with SC/P.

The results of redundancy analysis (RDA) (Figure 4d) indicated that the variance contribution rates of leaf functional traits in the first and second ordination axes were 42.99% and 20.44%, respectively (Figure 4d). Collectively, the first two axes cumulatively accounted for 63.43% of the leaf trait characteristics, suggesting that these axes effectively captured the relationship between leaf functional traits and environmental factors, with the first axis being the primary determinant. As depicted in the figure, environmental factors exerted differential influences on the variation in leaf functional traits, among which SAN, eC/N, SC/P, and BG were the main environmental factors affecting the variation in leaf functional traits (Figure 4d).

### 2.5. Effects of Soil Cover Thickness and Soil Factors on Plant Functional Traits

**Figure 5:**
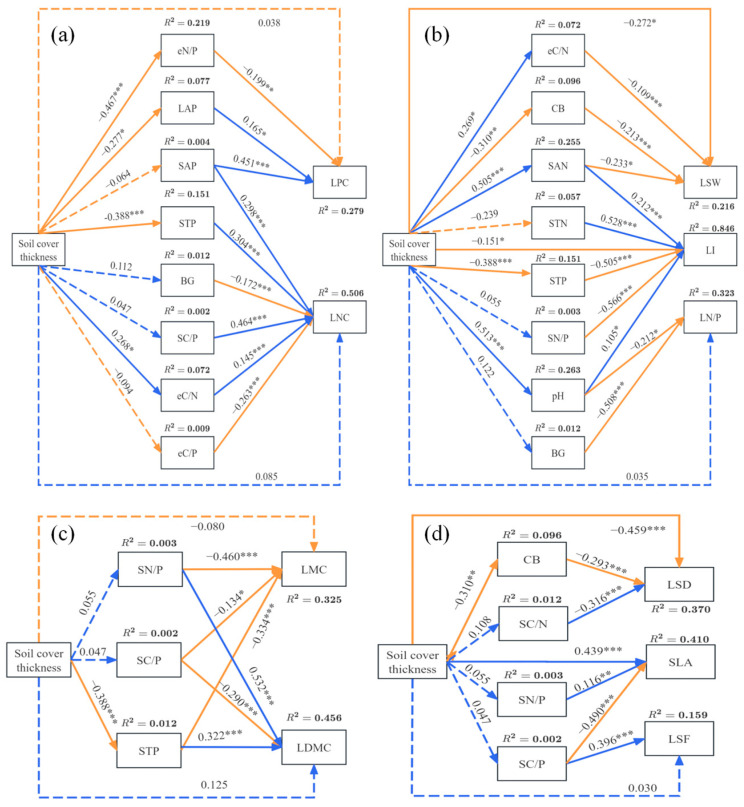
Structural equation models were employed to illustrate how soil cover thickness and soil factors affect leaf functional traits, both directly and indirectly. (**a**) LNC, Leaf nitrogen content; LPC, Leaf phosphorus content; (**b**) LSW, Leaf specific weight; LN/P, Leaf nitrogen/phosphorus; LI, Leaf shape index; (**c**) LMC, Leaf moisture content; LDMC, Leaf dry matter content; (**d**) LSF, Leaf shape factor; SLA, Specific leaf area; LSD, Leaf succulence degree. Solid blue arrows indicate significant positive effects, while solid orange arrows represent significant negative effects, and dotted blue and orange arrows denote non-significant paths. Path standardized coefficients (*β*) are marked on the arrows, and significance levels are categorized as follows: * *p* < 0.05; ** *p* < 0.01; *** *p* < 0.001. (For interpretation of the references to color in this figure legend, the reader is referred to the web version of this article.). SOC, Soil organic carbon; STN, Soil total nitrogen; STP, Soil total phosphorus; SMC, Soil moisture content; SAP, Soil available phosphorus; SAN, Soil ammonium nitrogen; SNN, Soil nitrate nitrogen; SC/N, Soil organic carbon/total nitrogen; SC/P, Soil organic carbon/total phosphorus; SN/P, Soil total nitrogen/total phosphorus; CB, Cellulase; BG, β-1,4-glucosidase; NAG, N-acetyl-β-D-glucosaminidase; LAP, Leucine aminopeptidase; AP, Acid phosphatase; eC/N, ln (BG + CB)/ln (NAG + LAP) enzyme C/N; eC/P, ln (BG + CB)/ln (AP) enzyme C/P; eN/P, ln (NAG + LAP)/ln (AP) enzyme N/P.

The SEM analysis indicated that soil cover thickness indirectly influenced plant functional traits by influencing soil characteristics (Figure 5). Our SEM explained 50.6%, 27.9%, 21.6%, 84.6%, 32.3%, 32.5%, 45.6%, 37.0%, 41.0%, and 15.9% of the variation in LNC, LPC, LSW, LI, LN/P, LMC, LDMC, LSD, SLA, and LSF, respectively (Figure 5). The SEM analysis identified the strongest correlations between soil cover thickness and soil pH (*β* = 0.513) and SAN (*β* = 0.505) (Figure 5). For LPC, LNC, LN/P, LMC, LDMC, and LSF, soil cover thickness did not directly affect them (Figure 5). The effect of soil cover thickness on LNC was mediated by STP and eC/N, where soil cover thickness indirectly increased LNC by lowering STP and increasing eC/N. The effect of soil cover thickness on LPC was mediated by LAP and eN/P (Figure 5a). Soil cover thickness had significant effects on SAN, eC/N, STP, and pH, while LSW was significantly negatively correlated with SAN and eC/N. LI was negatively correlated with STP and LN/P with pH, but LI was positively correlated with SAN and pH. Additionally, soil cover thickness directly and significantly affected LSW and LI (Figure 5b). The effect of soil cover thickness on LMC and LDMC was mediated indirectly through its effect on STP, with STP showing significantly negative and positive correlations with LPC and LDMC, respectively (Figure 5c). Similarly, soil cover thickness indirectly affected LSD significantly through CB, while it also had a direct effect on LSD and SLA (Figure 5d).

## 3. Discussion

### 3.1. Effect of Soil Cover Thickness on Leaf Functional Traits of Vines

The leaf economic spectrum provides a framework for coordinating and quantifying a series of leaf traits, thereby elucidating the trade-off between leaf structural costs and resource return time in plants [15]. According to the leaf economic spectrum theory, plants can be categorized into two types: “fast investment-return” (resource acquisition type) and “slow investment-return” (resource conservative type) [24]. In this study, we observed that across three different soil cover depths, the LMC, LNC, and SLA of *P. lobata* significantly exceeded those of the other five plant species. However, its LSW and LDMC were significantly lower compared to other woody vines. This indicated that *P. lobata* exhibited a resource acquisition type strategy, belonging to the “fast investment-return” plant category. By having thinner leaves, lower dry matter content, and higher water content, *P. lobata* rapidly expands its leaf area and enhances nutrient absorption, making it better suited to complex environmental resource conditions. Additionally, the leaf lifespan showed a significant positive correlation with LSW [25], suggesting that *P. lobata* had a relatively short leaf lifespan. By contrast, *H. nepalensis* exhibited a higher LSW and LDMC across all three soil cover depths, aligning with the traits of “slow investment-return” plants, indicating that it was a resource-conserving species.

The relative stability of plant functional traits and their plasticity to environmental changes enable plants to reflect their strategies and mechanisms for adaptation at different levels (from organs to species, communities, and ecosystems) [11]. The analysis of variance in this study showed that the responses of leaf functional traits of different vine plants to soil cover conditions exhibited significant species-specific and environment-dependent variations. Specifically, we found that *C. grandiflora* showed decreases in LMC, LI, and LSF with increasing soil cover depth, while LNC and LDMC increased. These changes indicated that *C. grandiflora* gradually shifted toward a more conservative resource allocation strategy with thicker soil layers. In deeper soil layers, *C. grandiflora* reduced its leaf water content and minimized investment in leaf morphology. This adaptation likely helped to reduce energy expenditure and promote dry matter accumulation in its leaves, thereby enhancing its resource-use efficiency under thicker soil cover conditions. By contrast, *M. sempervirens* showed increases in SLA and LDMC with deeper soil layers, while LMC, LSW, and LSD decreased. These traits suggested that *M. sempervirens* exhibited more “fast investment-return” type characteristics as soil cover depth increased. This may be due to the ample resources in the deeper soil layers, allowing the plant to reduce its demand for high metabolic activity and instead invest more in dry matter in leaves, optimizing resource use and shortening leaf lifespan to enhance resource use efficiency [26]. Furthermore, *P. tricuspidata* exhibited increasing trends in LNC, LPC, and SLA with greater soil cover depth, while LSD decreased, indicating that *P. tricuspidata* transitioned from a resource-conservative to a resource-acquisition strategy as soil cover depth increased. This shift may be attributed to the reduced reliance on thick mesophyll to cope with moisture and nutrient pressures, thereby conserving resources for enhancing leaf nitrogen and phosphorus content and specific leaf area to improve photosynthetic efficiency [27]. These adaptive mechanisms help vine plants maintain their growth and reproductive competitiveness in changing environments.

Gusewell [28] showed that when the leaf nitrogen to phosphorus ratio (LN/P) exceeds 16, plant growth is limited by phosphorus; when LN/P is below 14, nitrogen limits plant growth; and when LN/P is between 14 and 16, both nitrogen and phosphorus jointly limit growth. In this study, the LN/P ratios of the six vine plant species under different soil cover depths were all below 14, indicating that plant growth in the experimental area was nitrogen limited. In conclusion, this study highlighted the species-specific responses of leaf functional traits of vine plants to varying soil cover depths, as well as the differences in resource acquisition and conservation strategies among different species. Among them, according to their adaptation strategies, *P. tricuspidata* and *M. sempervirens* are more suitable for planting in thick soil layers, while *C*. *grandiflora* is suitable for planting in thin soil layers, and *P*. *lobata* and *H*. *nepalensis* are suitable for various soil cover thicknesses. Through these adaptive strategies, vine plants can effectively cope with changes in soil resource availability and optimize their growth and reproductive advantages.

### 3.2. Effects of Soil Cover Thickness on Soil Physical and Chemical Properties

This study found that the soil pH values under different vine species and soil cover thicknesses remained relatively stable, ranging from 4.71 to 5.27, indicating a weakly acidic environment. This suggested that both soil cover thickness and plant species have a minimal effect on soil pH, which may be attributed to the inherent buffering capacity of mining soil [29]. The pH and available nitrogen content of *P. quinquefolia*, *H. nepalensis*, and *C. grandiflora* increased with the thickness of the soil cover, indicating that these species might adapt to environmental changes by regulating soil pH and accelerating organic nitrogen mineralization to release more ammonium and nitrate under thicker soil cover conditions. Furthermore, this study found that the SMC of *P. quinquefolia* increased with increasing soil cover thickness, whereas *H. nepalensis* and *P. tricuspidata* exhibited the opposite trend, which suggested that the root distribution and water utilization strategies of different vine species vary. *P. quinquefolia*, with its developed root system, has a relatively low requirement for soil aeration; whereas, the shallow-rooted *H. nepalensis* and *P. tricuspidata* are more sensitive to soil aeration, and the increased cover thickness may have reduced their root vitality, impairing water absorption and affecting the soil SMC. Since *P. lobata* is a perennial leguminous vine with a higher nitrogen utilization rate, it exhibited a lower STN across all three soil cover thicknesses, with a gradual decrease in SNN as the soil cover thickness increased [30]. By contrast, *P. lobata* exhibited a higher STP content across different cover thicknesses, suggesting its efficient phosphorus acquisition and accumulation. Phosphorus is a limiting nutrient for plant growth [31], and the higher STP content may facilitate better growth of *P. lobata* in mining soils. Additionally, the study found that the soil under different vine species (*P. quinquefolia*, *C. grandiflora*, *M. sempervirens*, and *P. tricuspidata*) exhibited good nitrogen and phosphorus accumulation capabilities under various soil cover thicknesses. However, the nutrient accumulation patterns differed among these species, which may be intrinsically linked to their root characteristics and associations with soil microorganisms [32].

The C/P and N/P ratios of the soil reflect the nutrient balance status [33]. Among these, the soil carbon-to-phosphorus ratio (SC/P) is a critical indicator of the bioavailability of phosphorus, with a lower SC/P value indicating greater potential for soil microorganisms to release phosphorus during organic matter mineralization [34]. The results showed that *P. lobata* had a smaller SC/P across all three soil cover thicknesses, suggesting that soil microorganisms could effectively release phosphorus during organic matter mineralization, leading to higher soil available phosphorus (SAP), particularly under a 60 cm cover thickness. Finally, the nitrogen-to-phosphorus ratio (SN/P) serves as a limiting nutrient indicator for plant growth and can also be used to assess the nitrogen saturation state of the soil [35]. In this study, the SN/P ratio of the various vine species at different soil cover thicknesses was lower than the average value in China [36], indicating that nitrogen was the primary limiting factor for the growth of the vine species in this mining area. This finding aligns with the observed pattern of LN/P ratios in the vines, further confirming the dominant role of nitrogen in plant growth. In summary, the analysis of soil nutrient conditions under different soil cover thicknesses for various vine species provides valuable insights for optimizing soil management and plant restoration strategies in mining areas.

### 3.3. Effects of Soil Cover Thickness on Soil Enzyme Activities and Stoichiometric Characteristics

Soil enzyme activities are an important indicator of soil fertility, as they reflect the relationship between soil biological metabolic demands and the availability of nutrients [37]. In this study, we found that the soil enzyme activities in soils planted with different vine plants responded differently to changes in soil cover thickness. The possible reason for this is that soil layer thickness limits the root activity of plants and the metabolic activities of soil microorganisms, thus affecting soil enzyme activities. Specifically, the soil activities of CB, NAG, and LAP planted with *P. quinquefolia* decreased as the soil cover thickness increased, while the BG and AP activities increased with increasing cover thickness. This phenomenon suggested that a thicker soil layer provides ample simple carbon sources, nitrogen sources, and increased phosphorus mineralization, which in turn reduced CB, NAG, and LAP activities while enhancing BG and AP activities. Interestingly, the AP activity was consistently higher than other enzyme activities as the soil cover thickness increased. This result indicated that, in phosphorus-deficient subtropical regions, microorganisms predominantly produce phosphorus-acquiring enzymes to meet the phosphorus demand of plants [38]. Among the six vine plants tested, the AP activity at all three cover thicknesses in soils planted with *H. nepalensis* was significantly higher than that of the other vine plants, indicating that *H. nepalensis* has a higher demand for phosphorus in the soil. Additionally, the CB activity of *C. grandiflora* and the NAG activity of *P. tricuspidata* decreased with increasing soil cover thickness, suggesting that the facilitative effects of *C. grandiflora* on the soil carbon cycle and *P. tricuspidata* on the soil nitrogen cycle weakened with the increasing cover thickness. This could be related to the changes in the root system’s adaptation to soil aeration. By contrast, the LAP and AP activities of *M. sempervirens* increased significantly with increasing soil cover thickness, indicating that its facilitative effect on soil nitrogen and phosphorus cycles intensified with greater soil cover thickness. This may be due to its roots’ efficient absorption and utilization of soil nutrients.

The stoichiometric ratios of soil enzymes (e.g., eC/N, eC/P, and eN/P) reflect the cycling and transformation of elements such as carbon, nitrogen, and phosphorus within the soil matrix. These ratios can also elucidate microbial nutrient acquisition strategies and the availability of limiting resources [39]. Numerous previous studies have identified phosphorus as a primary constraint on ecosystem productivity in subtropical regions [34]. However, this study revealed that, at all three cover thicknesses, the eC/N ratios of soils planted with different vine plants were lower than the global average for terrestrial ecosystems (1.41), while the eN/P ratios were higher than the global soil eN/P ratio (0.44) [39]. This result suggests that soil microorganisms in this region tended to invest more in nitrogen-acquiring enzyme synthesis rather than phosphorus-acquiring enzyme synthesis. According to the resource allocation theory, when nitrogen is limiting in soil, microorganisms will prioritize the acquisition of nitrogen over phosphorus, thereby increasing resource allocation to nitrogen-acquiring enzymes, which subsequently enhances the activity of nitrogen hydrolases [37]. This indicates that, despite the phosphorus scarcity in subtropical regions, nitrogen limitation may, to some extent, dominate the metabolic strategy of soil microorganisms.

### 3.4. Trade-Offs and Synergies Between Plant Functional Traits

The coordination of plant functional traits often results from trade-offs in biophysical processes within a comprehensive ecological strategy [40]. In this study, SLA exhibited a significantly positive correlation with LNC and LN/P, indicating the photosynthetic capacity and nutrient turnover in vine plants. This suggested that a higher SLA is associated with greater photosynthetic ability, increased accumulation of photosynthetic products, and more rapid nutrient cycling, which aligns with the global leaf economic spectrum [24,41]. Conversely, SLA showed significantly negative correlations with LSW, LSD, and LDMC. This phenomenon may be due to the fact that leaves with a larger SLA are typically thinner and have a higher water content, leading to lower accumulations of LSD and LDMC. This observation aligns with the physiological strategy of plants optimizing photosynthesis and water use by increasing leaf area. Furthermore, LMC was significantly negatively correlated with LSW and LDMC, suggesting that a higher leaf water content may reflect a more flexible leaf structure that aids in water storage [42]. Consequently, in environments with sufficient water, plants may not require extensive dry matter accumulation, resulting in a relatively low LSW and LDMC. This study also identified a significantly negative correlation between LPC and LN/P, but no significantly negative correlation with LNC. This suggests that LN/P is primarily determined by phosphorus content, aligning with the findings of Duan et al. [43]. Additionally, a significantly positive correlation was observed between LSW and both LSD and LDMC. A higher LSW typically indicates that the leaf tissue is tougher and more fleshy, which enhances the plant’s adaptability to stress conditions such as drought, thus increasing dry matter accumulation [44]. Notably, no significant correlations were detected between LI and LSF with other functional traits. Although LI and LSF may be related to plant growth morphology, leaf distribution, or light utilization, their relationships with physiological functional traits (such as photosynthetic efficiency and leaf nutrient composition) may be more complex and difficult to reveal through simple correlation analysis. The principal component analysis (PCA) results were consistent with the Pearson’s correlation analysis, further validating the interrelationships among different functional traits and providing a reliable basis for multidimensional integrated analysis of plant functional traits.

### 3.5. Soil Cover Thickness Affects Plant Functional Traits Through Soil Factors

Research has shown that the functional traits of vine plants exhibit differential responses to soil factors, and the effect of soil cover thickness on these traits is also variable. The results of structural equation modeling (SEM) indicated that the impact of soil cover thickness on plant functional traits is primarily mediated indirectly through a series of soil factors. These findings suggested that changes in the soil environment, particularly variations in soil cover thickness, play an important regulatory role in plant functional traits. Soil pH variations directly affect root nutrient absorption efficiency and microbial activity, while SAN is a key nutrient for plant absorption and is closely related to soil pH [45]. Therefore, this study found a strong correlation between soil cover thickness and both soil pH (*β* = 0.513) and SAN (*β* = 0.505). Additionally, this research revealed that changes in soil cover thickness significantly reduced STP and increased eC/N, which in turn enhanced the leaf nitrogen content. This is because a high eC/N ratio reflects nitrogen limitation in microbial activity. When the soil phosphorus content decreases, plants tend to enhance nitrogen absorption or accelerate nitrogen metabolism to adapt to nutrient limitations, thereby maintaining growth and metabolic stability [46]. Phosphorus and nitrogen are two key nutrients for plant growth, and they function complementarily within plants [31].

Although soil cover thickness did not significantly affect SAP, BG, or eC/P, a significantly positive correlation was observed between SAP and LNC, while BG and eC/P were negatively correlated with LNC. Phosphorus enhances the root function and nitrogen absorption capacity of plants, which may lead to increased leaf nitrogen content [47]. Conversely, high BG enzyme activity and a high eC/P ratio typically indicate increased microbial activity, leading to higher nitrogen consumption in the soil [48], which reduces the nitrogen available to plants and decreases the leaf nitrogen content. In this study, soil cover thickness significantly negatively affected LAP and eN/P, but the reduction in LAP and the increase in eN/P promoted leaf phosphorus accumulation. This suggested that changes in soil cover thickness significantly reduce the secretion of nitrogen-acquiring enzymes in microorganisms while increasing the synthesis of phosphatases to acquire more phosphorus, thereby increasing the leaf phosphorus content in plants. Furthermore, although soil cover thickness did not affect SAP, SAP had a significantly positive effect on LPC, indicating that the supply of effective phosphorus increased the plant’s ability to absorb phosphorus through the roots, thereby enhancing the overall phosphorus content of the plant [49]. CB, SAN, and eC/N had significantly negative effects on LSW, suggesting that high CB and eC/N values indicate microbial preference for carbon source decomposition, leading to nitrogen deficiency. This, in turn, promotes the “cheap leaf” strategy in plants (low construction cost, high turnover rate), reducing leaf density and prioritizing resource allocation to the roots for greater nitrogen acquisition [50]. Soil cover thickness also indirectly influenced LMC and LDMC by altering STP. This indirect influence suggests that plants, when faced with phosphorus limitation, adjust their water and dry matter allocation strategies to optimize growth [24]. Notably, soil cover thickness directly had a significantly positive effect on SLA, while exerting significantly negative effects on LSW, LI, and LSD. This direct effect indicated that soil cover thickness not only influences plant morphological traits through changes in soil chemical properties but may also improve plant morphology directly, optimizing growth. In summary, soil cover thickness indirectly regulated plant functional traits by affecting most soil factors, especially through changes in soil nutrients (pH, STP, and SAN) and soil enzyme activities (CB, LAP, eC/N, and eN/P). However, soil cover thickness also directly affected a few plant functional traits. These findings not only enhance our understanding of soil–plant interactions but also provide important theoretical support for future ecological restoration and soil management efforts.

## 4. Materials and Methods

### 4.1. Site Description and Experimental Design

**Figure 6:**
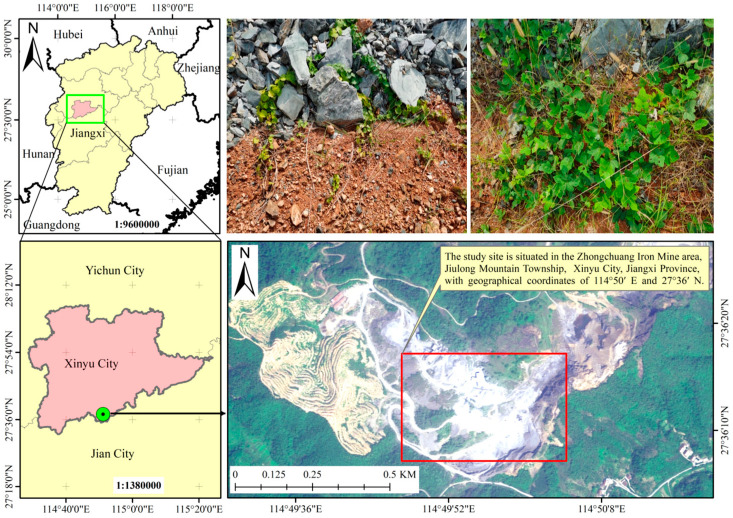
Map of the studied plots.

The study site is situated in the Zhongchuang Iron Mine area, Jiulong Mountain Township, Fairy Lake District, Xinyu City, Jiangxi Province, with geographical coordinates of 114°50′ E and 27°36′ N, at an elevation of 150.81 m (Figure 6). The region is characterized by a subtropical humid monsoon climate, with an average annual sunshine duration of approximately 1512 h, an average annual temperature of 17.5 °C, and an average annual precipitation of 1590.9 mm [51]. The annual reference evaporation is 1050 mm, the monthly peak value appears in July (6.8 mm/d), and the lowest value appears in January (1.2 mm/d). Precipitation is concentrated from April to June, with the highest rainfall occurring in June. The frost-free period lasts about 270 days. Climatic factor data were downloaded from https://data.cma.cn/market/index.html [Accessed 14 August 2024]. All data resolution is 1 km × 1 km. The parent material consists of phyllite, and the soil type is acrisols, with the majority of the soil layers exceeding 10 cm in thickness, which is favorable for the growth of vine plants [52]. Preliminary soil investigation data for the experimental site include: soil organic carbon content (SOC), 1.86 g·kg^−1^; soil total nitrogen content (STN), 0.23 g·kg^−1^; soil total phosphorus content (STP), 0.53 g·kg^−1^; soil moisture content (SMC), 22.61%; soil pH, 4.92; soil available phosphorus content (SAP), 1.76 mg·kg^−1^; soil ammonium nitrogen content (SAN), 3.42 mg·kg^−1^; and soil nitrate nitrogen content (SNN), 1.73 mg·kg^−1^. For the determination methods and references of these parameters, please see below.

In January 2023, the in situ soil backfill cover test was carried out at the bottom of the high and steep rock wall in the mining area of Xinyu City. The cover thicknesses were 20 cm, 40 cm, and 60 cm, respectively. The natural soil in the mining area without manual intervention or handling was backfilled and vibrated and compacted by machinery. The backfill size was a strip of width × length = 1 m × 20 m, and three repetition zones were set. In March 2023, six vine species (*Parthenocissus quinquefolia*, *Pueraria lobata*, *Hedera nepalensis*, *Campsis grandiflora, Mucuna sempervirens*, and *Parthenocissus tricuspidata*) were used for ecological restoration of the steep rock wall. Two-year-old container seedlings of the six vine species were transplanted into the three soil thicknesses, with 30 plants of each species per treatment, totaling 1620 plants.

### 4.2. Sample Collection

In August 2024, soil isometric sampling was carried out in the covering soil at the bottom of the high and steep rock wall in the mining area. During the sampling process, weeds and large stones on the soil surface were removed. For each treatment, 5 soil samples of the 0–20 cm soil layer were collected with a 70 mm diameter soil drill as composite samples. In total, 54 soil samples were collected and placed in disinfected self-sealing bags for pretreatment (visible vegetation, blanket debris, and gravel were removed using tweezers) and immediately transported back to the laboratory in an incubator. A portion of the soil samples was naturally air-dried, ground, and passed through 1 mm and 0.25 mm sieves to prepare for the analysis of available and total soil nutrients. Meanwhile, the remaining fresh soil samples were passed through a 2 mm sieve. To avoid clumping or clogging of the 2 mm mesh, the soil was gently homogenized before sieving, and then it was stored at −20 °C for subsequent enzyme activity assays. Prior to sampling, all tools, gloves, bags, and other sampling materials were thoroughly disinfected to prevent contamination.

### 4.3. Determination of Leaf Functional Traits

The fresh weight of 10 leaves from each individual plant was measured using an electronic balance. The leaves were subsequently positioned flat on a scanner (Epson Perfection V19) with the resolution adjusted to 300 dpi. The scanned images of the leaves were processed in batch using WinFOLIA LA-S leaf analysis software (Hangzhou Wanshen Detection Technology Co., Ltd., Hangzhou, China, www.wseen.com [Accessed 14 August 2024]), which calculated the leaf shape index (LI) and leaf shape factor (LSF) [53]. After scanning, the leaves were transferred to a drying oven maintained at 65 °C for a duration of 48 h and then weighed to determine their dry weight. The specific leaf area (SLA) and leaf specific dry weight (LSW) were calculated by combining the leaf dry weight and leaf area [54]. Additionally, the leaf moisture content (LMC) [55], leaf succulence degree (LSD), and leaf dry matter content (LDMC) were calculated by comparing the leaf dry weight and fresh weight [56]. Furthermore, a sample of dried leaves was taken to determine its nitrogen content (LNC) and phosphorus content (LPC), and the nitrogen-to-phosphorus ratio (LN/P) was subsequently calculated. The leaf nitrogen content (LNC) and leaf phosphorus content (LPC) were determined using the same method as for soil total nitrogen (STN) and soil total phosphorus (STP). Please see below for the specific methods. The specific calculation formulas are as follows:

Leaf Shape Index (LI) = Leaf length/Leaf width(1)

Leaf Shape Factor (LSF) = 4π × Leaf Area/Leaf Perimeter^2^ (2)

Specific Leaf Area (cm^2^·g) ((SLA) = Leaf Area/Leaf Dry Weight (3)

Leaf Specific Weight (10^3^·g·cm^−2^) (LSW) = Leaf Dry Weight/Leaf Area (4)

Leaf Succulence Degree (10^2^·g·cm^−2^) (LSD) = (Leaf Saturated Water Weight − Leaf Dry Weight)/Leaf Area (5)

Leaf Dry Matter Content (%) (LDC) = Leaf Dry Weight/Leaf Saturated Fresh Weight × 100%(6)

Leaf Moisture Content (%) (LMC) = (Leaf Fresh Weight − Leaf Dry Weight)/Leaf Fresh Weight × 100%(7)

Leaf Nitrogen-to-Phosphorus ratio (LN/P) = Leaf Nitrogen Content (LNC)/Leaf Phosphorus Content (LPC)(8)

### 4.4. Soil Chemical Properties Measurements

Soil pH was measured using a pH meter with a soil-to-water ratio of 1:2.5 (PHS-3C, INESA (Instruments and Electronics Associates, Shanghai, China) [57]. The soil moisture content (SMC) was determined by drying a soil sample at 105 °C for 48 h until it reached a constant weight and then calculating the moisture percentage based on the weight difference [58]. Soil organic carbon (SOC) was measured using a total organic carbon analyzer (Multi N/C 2100S, Analytic Jena, Jena, Germany), which quantified the carbon content after the soil samples were oxidized and converted into CO_2_ for detection [59]. The soil was digested with H_2_SO_4_−HClO_4_, and the total nitrogen (TN) and total phosphorus (TP) were determined using a fully automated chemical analyzer (Smartchem 200, ALLIANCE, Paris, France) [60]. The Olsen method was used to determine soil available phosphorus (SAP) by extracting soil with a 0.5 mol·L^−1^ NaHCO_3_ solution and measuring the phosphorus concentration in the extract using a spectrophotometer [61]. Soil available nitrogen (including soil ammonium nitrogen (SAN) and soil nitrate nitrogen (SNN)) was determined using 2 mol·L^−1^ KCl as the extraction solution (soil-to-solution ratio 1:10), followed by shaking at 180 rpm for 2 h, and then the nitrogen concentration was measured using a automatic discontinuous chemical analyzer (AMS SmartChem140, AMS-Alliance, Weston, FL, USA) [62].

### 4.5. Soil Enzyme Activity Measurements

The potential activities of various hydrolases, including cellulase (CB), β-1,4-glucosidase (BG), N-acetyl-β-D-glucosaminidase (NAG), leucine aminopeptidase (LAP), and acid phosphatase (AP), were evaluated in soil homogenates using the method described by German et al. [63] and Otgonsuren et al. [64]. Specifically, soil homogenates were prepared by dispersing 1 g of fresh soil in 100 mL of 100 mM sodium acetate buffer adjusted to pH 5.5. After sonicating for 1 min, 200 μL aliquots of the homogenate were transferred to 96-well plates under constant stirring, with each treatment replicated four times. Subsequently, 50 μL of the corresponding MUF- or AMC-labeled substrates (0.5 mM for CB, 1 mM for BG, 1 mM for NAG, 2 mM for AP, and 1 mM for LAP) were added to the wells, followed by a 2 h incubation at temperatures of 5, 10, 15, and 20 °C. Standard curves were established for both AMC (50 μM and 100 μM for LAP) and MUF (20 μM, 50 μM, 100 μM, and 250 μM for the other enzymes) to cover a broad range of fluorescence intensities. Fluorescence was immediately measured using a multimode plate reader (EnSpire) with an excitation wavelength of 365 nm and an emission wavelength of 460 nm. Enzyme activities were expressed as nmol of product released per g dry soil per hour. Additionally, the soil enzyme stoichiometric ratio was calculated based on the activities of hydrolases related to C, N, and P, following the methods outlined in [39,60]:

enzyme C/N activity ratio = In (BG + CB)/In (NAG + LAP)(9)

enzyme C/P activity ratio = In (BG + CB)/In (AP)(10)

enzyme N/P activity ratio = In (NAG + LAP)/In (AP)(11)

### 4.6. Statistical Analysis

We employed SPSS software (Version 26.0; IBM SPSS Statistics, Armonk, NY, USA) to conduct a one-way analysis of variance (ANOVA) followed by LSD post hoc tests for comparing differences in leaf functional traits and soil factors across different soil cover thicknesses. Pearson’s method and principal component analysis (PCA) were used to test the correlations, synergy, and trade-off relationships among multiple leaf traits. Concurrently, we analyzed the correlations between leaf functional traits and soil factors (OriginPro 2023; OriginLab Corporation, Northampton, MA, USA). Redundancy analysis (RDA) was conducted to identify the key environmental factors (soil thickness and soil factors) that significantly influence leaf functional traits (OriginPro 2023; OriginLab Corporation, Northampton, MA, USA). Subsequently, we constructed a structural equation model (SEM) [65] to examine the indirect effects of cover thickness on leaf functional traits via soil enzyme activities and nutrient cycling. For SEM, we utilized the R package (https://www.jstatsoft.org/article/view/v048i02) [Accessed 24 September 2024] “lavaan” [66]. We used “ggplot2” package [67] in R to plot the results.

## 5. Conclusions

This study, to some extent, quantified the relationships between trait–trait interactions and trait–environment linkages for six vine plant species under varying soil cover thicknesses in a mining area. The responses of leaf functional traits to varying soil cover thicknesses exhibited significant species-specific differences, with each vine plant species displaying distinct resource acquisition and conservation strategies. These strategies reflect the unique adaptive mechanisms employed by the plants in the mining environment. Among them, *P. lobata* demonstrated a resource acquisition strategy (fast investment-return), while *H. nepalensis* showed a resource conservation strategy (slow investment-return). As soil cover thickness increased, *C. grandiflora* and *P. tricuspidata* progressively adopted more conservative resource allocation strategies, whereas *M. sempervirens* exhibited the opposite trend. The study revealed that various leaf functional traits exhibited both synergistic relationships and trade-offs. Specifically, positive correlations were observed between SLA and LNC, LMC and LN/P, and LSW, LSD, and LDMC. Conversely, trade-offs were observed between SLA and LSW, LSD and LDMC, LPC and LN/P, as well as LMC, LSW, and LDMC. In terms of plant trait–environment relationships, soil factors such as nutrient content (pH, STP, and SAN) and soil enzyme activities (CB, LAP, eC/N, and eN/P) were the primary factors affecting the variations in leaf functional traits of vine plants. Interestingly, the nutrient ratios of plants, soil nutrients, and enzyme activity ratios suggested that nitrogen was the limiting factor for plant growth in the mining area. Overall, the responses of leaf functional traits of different vines under different soil thicknesses have significant species specificity, and each vine shows different resource acquisition and conservation strategies. In addition, soil cover thickness mainly influenced plant functional traits by directly affecting soil enzyme activities and nutrient availability, although the pathways of influence on different functional traits were not identical. This underscores the intricate interplay among soil cover thickness, soil properties, and plant functional traits. The results provide guidance for soil management and plant restoration strategies, particularly in addressing soil resource limitations and enhancing plant growth and reproductive capacity. Furthermore, the study validates the rationale and necessity of soil covering technology as an effective means for ecological restoration in mining areas with high and steep rock walls.

## Data Availability

Data from this study are available upon reasonable request from the corresponding author.
